# Discordant Predictions of Extraglandular Involvement in Primary Sjögren’s Syndrome According to the Anti-SSA/Ro60 Antibodies Detection Assay in a Cohort Study

**DOI:** 10.3390/jcm11010242

**Published:** 2022-01-04

**Authors:** Geoffrey Urbanski, Aline Gury, Pascale Jeannin, Alain Chevailler, Pierre Lozac’h, Pascal Reynier, Christian Lavigne, Carole Lacout, Emeline Vinatier

**Affiliations:** 1 Department of Internal Medicine and Clinical Immunology, University Hospital, 49000 Angers, France; aline.gury@gmail.com (A.G.); pierre.lozac@gmail.com (P.L.); ChLavigne@chu-angers.fr (C.L.); Carole.Lacout@chu-angers.fr (C.L.); 2 Mitolab, MitoVasc Institute, CNRS 6015, INSERM U1083, University of Angers, 49000 Angers, France; pareynier@chu-angers.fr; 3 Laboratory of Immunology, University Hospital, 49000 Angers, France; pascale.jeannin@univ-angers.fr (P.J.); chevailler.a@wanadoo.fr (A.C.); Emeline.Vinatier@chu-angers.fr (E.V.); 4 INSERM, CRCINA, Angers University, 49000 Angers, France; 5 Department of Internal Medicine, General Hospital, 72000 Le Mans, France

**Keywords:** primary Sjögren’s syndrome, anti-SSA antibodies, detection methods

## Abstract

Electrophoresis-derived techniques for anti-SSA/Ro60 KDa (anti-SSA) antibodies detection have been progressively replaced by methods using non-native antigens. We aimed to compare the patients’ phenotypes and the occurrence of extraglandular manifestations in primary Sjögren’s syndrome according to the method used to detect anti-SSA antibodies. Sera from patients with a diagnosis of pSS according to ACR/EULAR 2016 criteria between 2008 and 2017 were tested for anti-SSA antibodies using methods with non-native antigens (magnetic bead multiplex assay; line immunoassays) and one with native antigens (counterimmunoelectrophoresis (CIE)). The population was split into three groups according to anti-SSA antibodies status: absence (SSA−), presence in any method except for CIE (SSA+CIE−), and presence in CIE (SSA+CIE+). The patients in the SSA+CIE+ group (*n* = 70, 42.7%) were ten years younger and presented more immunological activity compared with both the SSA− (*n* = 80, 48.8%) and SSA+CIE− groups (*n* = 14, 8.5%). The SSA− and SSA+CIE− groups were poorly distinct. The presence of anti-SSA antibodies solely in CIE was significantly associated with the occurrence of extraglandular manifestations of pSS (HR = 4.45 (2.35–8.42)). Contrary to CIE, methods using non-native antigens to detect anti-SSA antibodies were unable to predict the occurrence of systemic expression of pSS.

## 1. Introduction

Anti-SSA/Ro60 (anti-SSA) antibodies represent a cornerstone in primary Sjögren’s syndrome (pSS). Even if they are not a mandatory criterion for the pSS diagnosis, they are present in 39% to 73% of patients from large cohorts depending on recruitment [1,2,3,4]. Originally described in 1961 by Anderson and colleagues as SjD antibodies [5], anti-SSA autoantibodies were clearly identified in the sera of patients with primary Sjögren’s syndrome using immunodiffusion in 1975 [6]. Even though anti-SSA antibodies are not fully sensitive and specific for pSS, they are an interesting characteristic for studying the pathogenesis of this disease as they appear many years before the first symptoms [7]. Thus, anti-SSA antibodies are a criterion in classification consensus, including the actual ACR/EULAR 2016 consensus [8]. Beyond their use as a diagnostic criteria, the presence of anti-SSA antibodies has been associated with early onset disease, more signs of B cell activity (hypergammaglobulinemia, rheumatoid factors, cryoglobulinemia, and naive/memory B cell imbalance), and more extraglandular manifestations in pSS [7,9,10,11,12,13]: Raynaud phenomenon [14], interstitial pneumonia [15], peripheral nerve involvement [14,16,17,18], cutaneous vasculitis [19,20,21,22], cytopenias [16,19,23,24], adenopathies [14,19,25], and lymphoma [12,25,26].

Former gold standard techniques for detecting anti-SSA antibodies were RNA precipitation, double-immunodiffusion, counterimmunoelectrophoresis (CIE), immunoprecipitation, and Western blotting [27]. Among these, CIE seemed to present the best balance between performances and technical constraints [28], as it was quite rapid and able to detect a small concentration of antibody. Moreover, such a method mimics the natural interaction between antigens and antibodies, highlighting autoantibodies able to precipitate (visualised with line of precipitation) [6]. This homemade method was progressively replaced by enzyme-linked immunosorbent assay (ELISA) and line immunoassay (LIA) for cost and time effectiveness reasons [29]. The current widespread method is the automatic multiplex immunoassay using magnetic beads covered with antigens, allowing detection of many antinuclear antibodies quickly and with a low sample volume [30].

To our knowledge, most studies comparing the predictive value of anti-SSA antibodies in pSS did not differentiate between these methods in the interpretation of their results. This could be troubling due to the different analytical conditions of the immunoassays. Indeed, recent and widespread methods (LIA, ELISA, and multiplex assays) use purified or recombinant proteins that are denatured through preparation process or during a coating step, whereas epitopes recognised by anti-SSA antibodies are highly conformational and the binding is often lost with denaturation [31,32,33]. Identifying antibodies with recombinant or denatured protein raises questions about the ability to reflect the pathological process of the disease and the probability of a false positive [34].

pSS is associated with extraglandular manifestations that reflect the systemic activity of the disease. These manifestations are of interest because they represent the severity of pSS. Identifying markers to predict the occurrence of extraglandular manifestations is thus a major concern.

This study aimed to compare the onset of extraglandular involvement in pSS patients according to the absence or presence of anti-SSA antibodies using three methods of detection: CIE, LIA, and multiplex assays.

## 2. Patients and Methods

### 2.1. Ethics

The study was approved by the Ethical Committees of Angers University Hospital (*n*° 2018/55) and was conducted in compliance with the declaration of Helsinki. All participants gave non-opposition informed consent. This study applied the Strengthening the Reporting of Observational Studies in Epidemiology (STROBE) statement to observational studies.

### 2.2. Inclusion and Exclusion Criteria

We extensively revised the files of patients aged 18 years and over who were referred on suspicion of Sjögren’s syndrome in our Internal Medicine Department between January 2008 and December 2017. We included patients with primary Sjögren’s syndrome (pSS) according to ACR/EULAR 2016 criteria [8]. We excluded patients with no available serum samples that had been collected during the 24 months following pSS diagnosis. We also excluded patients with secondary Sjögren’s syndrome and/or with a follow-up of less than 12 months.

### 2.3. Data Collection

We extracted the following data: age at diagnosis, sex, follow-up duration, presence of eye and/or mouth dryness, results from Schirmer I test and unstimulated whole saliva (UWS) flow rate, results from minor salivary gland biopsy (MSGB), presence of antinuclear antibodies (on HEp-2 cells), anti-SSA, anti-SSB antibodies, rheumatoid factors, cryoglobulinemia, C3 and C4 fractions of complement, hypergammaglobulinemia defined as a blood gammaglobuline level of over 15 g/L, and extraglandular manifestations, as detailed below.

### 2.4. Definition of Extraglandular Manifestations

We considered extraglandular manifestations to be all measurable items listed in the ESSDAI (European League Against Rheumatism Sjögren’s Syndrome Disease Activity Index) score [35] except for constitutional, glandular and biological items. We also excluded non-objective signs, i.e., arthralgia without arthritis and cough. For cytopenia, we only considered a clinically significant level of cytopenia, i.e., a moderate and high degree of activity from ESSDAI (haemoglobin ≤100 g/L; platelets ≤100,000/mm^3^; lymphocytes ≤500/mm^3^; neutrophils ≤1000/mm^3^). All these extraglandular manifestations were considered related to pSS after checking for the exclusion of differential diagnoses, notably for cytopenias. We also considered interstitial cystitis to be an extraglandular manifestation related to pSS [36], even if not listed in ESSDAI. Raynaud phenomenon was not considered to be an extraglandular manifestation. We collected data about small fibre neuropathy but it was treated separately because of its association with the anti-SSA negative form of pSS [18,37,38].

Extraglandular manifestations were split into those occurring before pSS diagnosis and those occurring after.

### 2.5. Detection of Anti-SSA Antibodies

We used serum samples frozen at −20 °C and collected during the 24 months following pSS diagnosis. They were all tested for each participant at the same time using all four techniques employed by this study. The presence of anti-SSA60 kDa/Ro (anti-SSA) antibodies was tested for using (i) magnetic bead multiplex assay (Bioplex^®^ 2200, ANA screen kit, Biorad, Hercules, CA, USA), (ii) LIA Fullana Dot^®^ (Alphadia, Mons, Belgium) with automated reading on BlueDiver^®^ (Alphadia, Mons, Belgium), and (iii) LIA Inno-Lia ANA^®^ (Fujirebio, Tokyo, Japan) with manual reading. The fourth assay was CIE, adapted from a previously described method [39]. Briefly, we realised an indubiose film at a pH of 8.2 with barbital buffer. The indubiose plates were dug out of 2 columns of 15 wells in order to alternately deposit test and control sera, and of 2 troughs 3 mm wide cut parallel to the wells, 1 filled with primate spleen extract and 1 filled with rabbit thymus extract. Electrophoresis was carried out at 12 mA/slide for 90 min in barbital buffer. Precipitins were identified 24 h after the electrophoresis by at least 2 expert readers (A.G., A.C., and C.L. (Carole Lacout) among authors and G.R. in acknowledgements) detecting precipitation lines of identity with reference sera in adjacent wells.

We did not consider in this study anti-TRIM21 antibodies (previously named anti-SSA52 kDa) as their target differs from anti-SSA antibodies [40], and as they are not associated with pSS [41].

### 2.6. Constitution of Groups

The whole population was separated in three groups according to the results of anti-SSA antibodies detection: patients without anti-SSA antibodies (SSA−), patients with anti-SSA antibodies detected in multiplex and/or LIA but not in CIE (SSA+CIE−), and patients with anti-SSA antibodies detected in CIE whatever the results in multiplex and/or solid phase dots (SSA+CIE+).

### 2.7. Statistics

The quantitative data were presented in medians and quartiles and compared using a one-way ANOVA or Kruskal–Wallis test as appropriate. The categorical data were presented as absolute values and as percentages and were compared using a chi-squared test. The reliability between the tests for anti-SSA antibodies detection was evaluated by means of Cohen’s kappa coefficient.

Time-to-event curves for first incident extraglandular manifestations were presented as Kaplan–Meier curves and were compared with a log-rank test. Follow-up was limited at 120 months.

The influence of covariates on the occurrence of extraglandular manifestation was evaluated with a Cox model. The proportional hazard assumption was checked using 2 different methods: graphically by plotting the log(minuslog) curves and by studying the interaction with time. The alpha risk was 5%. The hazard ratios (HR) were presented with a confidence interval of 95%. The analyses were carried out using Graphpad Prism v6.01 (GraphPad Software, La Jolla, CA, USA) and SPSS software v23.0 (IBM Corp, Chicago, IL, USA).

## 3. Results

### 3.1. General Characteristics

From the 310 fully revised files between January 2008 and December 2017, 146 patients were excluded: 88 patients did not fulfil the ACR/EULAR 2016 criteria, 34 patients had secondary Sjögren’s syndrome, no serum sample was available for 18 patients, and 6 were excluded because of a follow-up time of less than 12 months. The study population included 164 patients with a median age of 59 (46–70) years and was composed of 140 (85.3%) females. Anti-SSA antibodies were detected in 84 (51.2%) patients, whatever the detection assay. The SSA−, SSA+CIE−, and SSA+CIE+ groups consisted of 80 (48.8%), 14 (8.5%), and 70 (42.7%) patients, respectively. There was no patient with sole positivity for anti-SSA antibodies in the solid phase dots (i.e., positive for one or two of them, and negative for multiplex and for CIE) that could define a specific group. The characteristics of the three groups are detailed in Table 1.

The SSA+CIE+ group was composed of younger patients compared with the two other groups (*p* = 0.008), with these patients being around ten years younger. They also displayed more immunological signs of activity (presence of antinuclear antibodies, hypergammaglobulinemia, rheumatoid factors, and cryoglobulinemia) compared with the SSA− and SSA+CIE− groups.

We chose not to compare the frequency of a focus score of ≥ 1 on the MSGB between each group as it was necessarily 100% in the SSA− group. However, it is important to note that 6/14 (42.9%) patients from the SSA+CIE− group had a focus score of < 1 on the MSGB, compared with 6/70 (8.6%) in the SSA+CIE+ group.

The type of extraglandular manifestations occurring before pSS diagnosis did not differ between the three groups. However, even in the absence of statistical difference, inaugural extraglandular manifestations were slightly more frequent in the SSA− group (Table 1). In contrast, patients from the SSA+CIE+ group declared inflammatory arthralgia more frequently.

### 3.2. Detection of Anti-SSA Antibodies

All patients from the SSA+CIE− group had anti-SSA antibodies detected in multiplex contrary to the SSA+CIE+ group in which 4 (5.7%) patients were negative for multiplex. Those four patients were also negative for both LIA.

The reliability between the two LIA themselves was poor (kappa = 0.125) for the SSA+CIE− group and good (kappa = 0.62) for the SSA+CIE+ group. In the SSA+CIE+ group, multiplex had good (kappa = 0.706) and moderate (kappa = 0.491) reliability with the LIA Fullana Dot^®^ and the LIA Inno-Lia ANA^®^ dots, respectively.

### 3.3. Occurrence of Extraglandular Manifestations after pSS Diagnosis

The characteristics of extraglandular manifestations occurring after pSS diagnosis are detailed in Table 2. Cytopenia, skin, and muscle involvements were more frequent in the SSA+CIE+ group.

**Figure 1:**
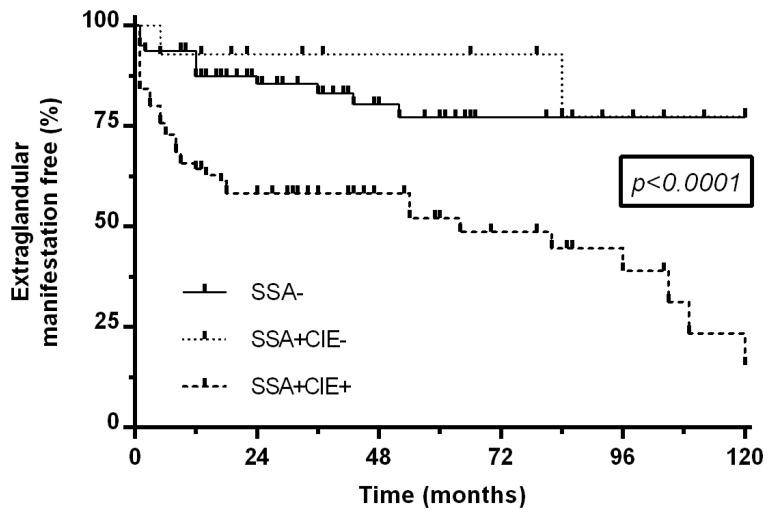
Extraglandular manifestations occurring after diagnosis in the 3 groups. Notes: the SSA− group referred to the patients with no anti-SSA antibodies. The SSA+CIE− referred to the patients with anti-SSA antibodies in any techniques except for counterimmunoelectrophoresis. The SSA+CIE+ referred to the patients with anti-SSA antibodies in counterimmunoelectrophoresis. CIE: counterimmunoelectrophoresis. The *p*-value on the graph represents the results of the comparison between the three curves using the log-rank test.

On the curves for extraglandular manifestations-free survival, the SSA+CIE+ group significantly differed from the two other groups (*p* < 0.0001, Figure 1), whereas there was no difference between the SSA− and SSA+CIE− groups (*p* = 0.58). In the Cox regression model, age (HR = 1.03 (1.01–1.05)) and especially the presence of anti-SSA antibodies in CIE (HR = 4.45 (2.35–8.42)) were significantly associated with the occurrence of extraglandular manifestations (Table 3).

### 3.4. Details inside the Groups

In the SSA+CIE− group, 6/14 (42.9%) patients did not present a focus score of ≥1 on the MSGB. None of those 6 patients had extraglandular manifestations either before or after the diagnosis (Supplemental Figure S1). They also did not present inflammatory arthralgia and small fibre neuropathy. Those 6 patients also had no B lymphocyte signs of hyperactivity (hypergammaglobulinemia, rheumatoid factors, low C3 or C4, cryoglobulinemia) except 1 patient, presenting antinuclear antibodies with a titer of 1/640.

Among the SSA+CIE+ group, 4/70 (5.7%) patients had positive anti-SSA antibodies detected only with CIE (i.e., negative for multiplex and dots assays), with three patients having extraglandular manifestations (that appeared before or after the diagnosis for one and two patients, respectively), with a median follow-up time of 36.5 [23–65.3] months. The fourth patient without extraglandular manifestation suffered from inflammatory arthralgia.

## 4. Discussion

With 85% females, a median age at diagnosis of 56 years, and 51% patients presenting anti-SSA antibodies, the general characteristics of our study population were consistent with the characteristics of published large cohorts [1,2,3,4]. Including all extraglandular manifestations (before and after diagnosis), 96/164 (58.5%) patients presented at least one extraglandular involvement in our whole population. This rate is quite similar to the cohorts of Baldini et al. (46.6%) [3] and Seror et al. (69.6%) [2], with minor differences, particularly less joint involvement compared with teams with rheumatologic department recruitment.

Anti-SSA antibodies have been historically associated with pSS on the basis of a detection method that uses the native structure of the antigen and highlights autoantibodies able to precipitate (visualised with line of precipitation) [6]. Since then, detection methods have evolved using non-native antigens for cost and time effectiveness reasons. However, the epitopes recognised by anti-SSA antibodies are highly conformational [31,32,33] and little is known about the consequences in clinical expression of detecting autoantibodies which recognise non-native antigens. In this study, we demonstrated that patients with anti-SSA antibodies detected using only denaturing methods were not so rare, concerning 14/84 (16.7%) of those having anti-SSA antibodies, and that they presented a significantly reduced extraglandular expression of pSS compared with those with anti-SSA antibodies detected using a precipitating method.

Over the past three decades, anti-SSA antibodies have been largely associated with extraglandular involvement in pSS [7,9,10,11,12,13]. Sandhya et al. noticed an odds ratio of 2.67 [1.09–6.54] of having an extraglandular manifestation in pSS patients presenting anti-SSA antibodies [42]. However, for most of these studies, the methods for assessing the anti-SSA antibodies are not specified and, for the few including it in the methods part, a single method was used. With the present study, we have demonstrated that the type of techniques used to detect anti-SSA antibodies, whether using native or denatured antigens, had an important impact on the ability to predict the systemic expression of pSS. Indeed, patients from the group with anti-SSA antibodies not detected in CIE had a weak pSS systemic expression, similar to those without anti-SSA antibodies (HR = 0.59 [0.13–2.66]). They also shared general characteristics, notably a close age at diagnosis, and were more than ten years older than patients with positivity on CIE; whereas, it is known in the literature that patients with anti-SSA antibodies are around 10 years younger compared with those without these antibodies [7,9,10,11,12,13]. Patients from the SSA+CIE− group clearly presented less frequent signs of B lymphocyte hyperactivity compared with patients with positive CIE. To summarise, the clinical and biological phenotypes of the patients with anti-SSA antibodies for only denaturing methods appeared as if they had no anti-SSA antibodies. Scofield et al. already noticed that the presence of neuropathy in pSS was associated with anti-SSA antibodies when determined using double immunodiffusion, another method that uses native antigens, but not when determined by ELISA [43]. To the best of our knowledge, our study is the first to highlight such findings for extraglandular involvements globally.

We demonstrated that patients with anti-SSA antibodies, which were undetectable with CIE, had a weak pSS systemic expression and were comparable to the SSA− group. This would first lead one to hypothesise the identification of a not yet pathogenic autoantibody in the case of latent autoimmune disease. However, when we detailed the composition of this group of patients, we noticed that 6/14 (43%) did not present significant sialadenitis on the MSGB, although they present sicca syndrome. This means that, without anti-SSA antibodies detection by multiplex and/or dots, those patients would not fulfil the criteria for pSS according to the ACR/EULAR consensus [1]. Moreover, none of these patients had extraglandular manifestations and swelling of the exocrine glands, whether before or after the diagnosis of pSS. These findings raise questions about a false positivity for these 6 patients among the 84 (7.1%) with anti-SSA antibodies. If the inability of anti-SSA antibodies detected using denaturing methods to predict the systemic expression of pSS is an interesting finding, such a false positive rate represents a more problematic question.

On the other hand, 4/84 (4.8%) patients presented anti-SSA antibodies only detected on CIE and all had a systemic expression of pSS. This confirms that denaturing methods failed to detect anti-SSA antibodies in some patients with a significant expression of pSS [44].

Among the four methods of detection we used, the main cleavage appeared between multiplex and CIE. The solid phase dots did not seem to define a third group compared with multiplex and CIE. Indeed, among patients with possible false positivity (presence of anti-SSA antibodies but negative on CIE and no significant sialadenitis), dots were positive for 5 patients out of 6.

One limitation of our study is the absence of ELISA, another common method to detect anti-SSA antibodies. However, some authors reported similar results to ours when using those assays, identifying a profile that did not correlate with the patient’s clinical presentation [45,46] and a significant false positivity rate for ELISA (5/12, 41.7%) compared with double immunodiffusion and immunoprecipitation [47].

A second limitation of our study could be the definition of extraglandular manifestations, which differed slightly from ESSDAI, for example. However, we thought it important to only consider objective and significant involvements in order to represent the systemic activity of pSS as clinically relevant. For example, the clinical significance of thrombopenia between 100,000 and 150,000/mm^3^ is limited. We also chose to exclude small fibre neuropathy from extraglandular manifestations because it has been associated with the profile of patients without anti-SSA antibodies [18,37,38] and it constituted a potential bias by overexpressing systemic involvement with this sole neurologic feature. The third limitation is the retrospective design of the study. We tried to correct this point by testing sera collected up to a maximum of 24 months following diagnosis and by analyzing all samples at the same time. Data collection was retrospective, but we have well-structured clinical activity concerning pSS with standardised procedures that minimise this bias. Finally, we have preferred to separate the manifestations occurring before and after pSS diagnosis because it is difficult to precisely define a starting point of the disease in pSS. Moreover, considering involvement before pSS diagnosis would have induced a bias of overestimation of events in patients presenting with extraglandular involvement compared with those presenting with sicca syndrome, possibly resulting in imbalance within the groups.

## 5. Conclusions

In this study, we highlighted that the method used to detect anti-SSA antibodies is a major concern. Methods using denatured antigens were unable to predict the occurrence of systemic expression of pSS contrary to CIE. Patients with anti-SSA antibodies that were undetectable with CIE (no line of precipitation) did not significantly differ from patients without anti-SSA antibodies. Moreover, our study suggests a potential false positivity for the diagnosis of pSS in patients without significant sialadenitis and with non-precipitating anti-SSA antibodies (only identified using denaturing methods). Our results raise questions regarding the value of using only non-native methods to detect anti-SSA antibodies in pSS.

## Figures and Tables

**Table 1:** General characteristics of patients from the 3 groups and detailed listing of extraglandular manifestations known before pSS diagnosis.

Groups	SSA−	SSA+CIE−	SSA+CIE+	*p*-Value
Number of patients	80	14	70	
Sex (female) *n (*%)	64 (80%)	13 (92.9%)	63 (90%)	0.16
Age at diagnosis (years)	61 (50.8–71)	64.5 (43.3–67.8)	52 (41.3–66)	0.008
Follow-up duration (months)	37.5 (17.8–67)	99.5 (33.8–120)	47 (30.3–120)	0.01
Subjective dry eye syndrome *n (*%)	70 (87.8%)	11 (78.6%)	59 (84.3%)	0.65
Subjective dry mouth syndrome *n (*%)	75 (93.8%)	13 (92.9%)	60 (85.7%)	0.24
ACR/EULAR 2016 criteria *n (*%)				
Schirmer I test ≤5 mm/5 min	68 (85%)	9 (64.3%)	41 (58.6%)	0.001
UWS flow rate ≤1.5 mL/15 min	41 (51.3%)	2 (14.3%)	33 (47.1%)	0.04
Anti-SSA antibodies	0 (0%)	14 (100%)	70 (100%)	-
Focus score ≥1 on Minor Salivary Gland Biopsy	80 (100%)	8 (57.1%)	64 (91.4%)	-
Other immunological features *n (*%)				
Anti-SSB antibodies	4 (5%)	3 (21.4%)	49 (70%)	<0.0001
Antinuclear antibodies titer ≥1/320	21 (26.3%)	6 (42.9%)	62 (88.6%)	<0.0001
Hypergammaglobulinemia (over 15 g/L)	5 (6.3%)	2 (14.3%)	31 (44.3%)	<0.0001
Presence of rheumatoid factors	12 (15%)	2 (14.3%)	45 (64.3%)	<0.0001
Decreased level of C3 and/or C4	12 (15%)	0 (0%)	17 (24.3%)	0.06
Cryoglobulinemia	9 (11.3%)	0 (0%)	17 (24.3%)	0.02
Detection of anti-SSA antibodies *n (*%)				
Multiplex	0 (0%)	14 (100%)	66 (94.3%)	-
LIA (Fullana Dot^®^)	0 (0%)	12 (85.7%)	63 (90%)	-
LIA (Inno-Lia ANA^®^)	0 (0%)	4 (28.6%)	59 (84.3%)	-
CIE	0 (0%)	0 (0%)	70 (100%)	-
Extraglandular manifestations occurring before the pSS diagnosis *n (*%)	12 (15%)	0 (0%)	6 (8.6%)	0.18
Extraglandular manifestations according to ESSDAI				
Lymphadenopathy and/or splenomegaly	0 (0%)	0 (0%)	1 (1.4%)	0.51
Lymphoma	0 (0%)	0 (0%)	0 (0%)	>0.99
Arthritis	0 (0%)	0 (0%)	2 (2.9%)	0.26
Skin	1 (1.3%)	0 (0%)	0 (0%)	0.59
Lung	3 (3.8%)	0 (0%)	2 (2.9%)	0.75
Kidney	0 (0%)	0 (0%)	0 (0%)	>0.99
Muscle	0 (0%)	0 (0%)	0 (0%)	>0.99
Peripheral nervous system	6 (7.5%)	0 (0%)	1 (1.4%)	0.13
Central nervous system	0 (0%)	0 (0%)	1 (1.4%)	0.51
Cytopenia	2 (2.5%)	0 (0%)	1 (1.4%)	0.77
Anaemia	1 (1.3%)	0 (0%)	1 (1.4%)	0.91
Thrombopenia	1 (1.3%)	0 (0%)	0 (0%)	0.59
Lymphopenia	0 (0%)	0 (0%)	0 (0%)	>0.99
Neutropenia	0 (0%)	0 (0%)	0 (0%)	>0.99
Extraglandular manifestations not listed in ESSDAI				
Interstitial cystitis	1 (1.3%)	0 (0%)	0 (0%)	0.59
Other manifestations related to pSS (before or after diagnosis)				
Small fibre neuropathy	11 (13.8%)	0 (0%)	2 (2.9%)	0.02
Enlarged parotid and/or lachrymal/submandibular gland swelling	10 (12.5%)	1 (7.1%)	10 (14.3%)	0.76
Arthralgia with morning stiffness over 30 min	17 (21.3%)	3 (21.4%)	28 (40%)	0.03

Notes: Cytopenia as listed in ESSDAI for score ‘moderate’ and ‘high’: haemoglobin ≤100 g/L; platelets ≤100,000/mm^3^; lymphocytes ≤500/mm^3^; neutrophils ≤1000/mm^3^. Continuous variables are presented with median and quartiles. CIE: counterimmunoelectrophoresis. ESSDAI: EULAR Sjögren’s syndrome disease activity index. LIA: line immunoassay. pSS: primary Sjögren’s syndrome. UWS: unstimulated whole saliva.

**Table 2:** Details of extraglandular manifestations occurring after pSS diagnosis.

Groups	SSA−	SSA+CIE−	SSA+CIE+	*p*-Value
Number of patients	80	14	70	
Extraglandular manifestations occurring after the pSS diagnosis *n (*%)	14 (17.5%)	2 (14.3%)	37 (52.9%)	<0.0001
Extraglandular manifestations according ESSDAI				
Lymphadenopathy and/or splenomegaly	0 (0%)	0 (0%)	4 (5.7%)	0.06
Lymphoma	2 (2.5%)	0 (0%)	1 (1.4%)	0.77
Arthritis	0 (0%)	0 (0%)	4 (5.7%)	0.06
Skin	1 (1.3%)	0 (0%)	10 (14.3%)	0.004
Lung	7 (8.8%)	1 (7.1%)	9 (12.9%)	0.65
Kidney	1 (1.3%)	0 (0%)	2 (2.9%)	0.66
Muscle	0 (0%)	0 (0%)	5 (7.1%)	0.03
Peripheral nervous system	6 (7.5%)	1 (7.1%)	5 (7.1%)	>0.99
Central nervous system	0 (0%)	0 (0%)	1 (1.4%)	0.51
Cytopenia	3 (3.8%)	0 (0%)	12 (17.1%)	0.008
Anaemia	1 (1.3%)	0 (0%)	5 (7.1%)	0.12
Thrombopenia	2 (2.5%)	0 (0%)	6 (8.6%)	0.15
Lymphopenia	0 (0%)	0 (0%)	1 (1.4%)	0.51
Neutropenia	0 (0%)	0 (0%)	2 (2.9%)	0.26
Extraglandular manifestations not listed in ESSDAI				
Interstitial cystitis	1 (1.3%)	0 (0%)	0 (0%)	0.59

Notes: Cytopenia as listed in ESSDAI for score ‘moderate’ and ‘high’: haemoglobin ≤100 g/L; platelets ≤100,000/mm^3^; lymphocytes ≤500/mm^3^; neutrophils ≤1000/mm^3^. Continuous variables are presented with median and quartiles. CIE: counterimmunoelectrophoresis. ESSDAI: EULAR Sjögren’s syndrome disease activity index. pSS: primary Sjögren’s syndrome. UWS: unstimulated whole saliva.

**Table 3:** Strength of association between incident extraglandular manifestations and anti-SSA antibodies status.

	Hazard Ratio (95%CI)	*p*-Value
Anti-SSA antibodies status		
SSA−	Reference	
SSA+CIE−	0.59 (0.13–2.66)	0.49
SSA+CIE+	4.45 (2.35–8.42)	0.000005
Age ^a^	1.03 (1.01–1.05)	0.002
Sex (female)	0.90 (0.38–2.15)	0.82
Presence of extraglandular manifestations before the pSS diagnosis	1.39 (0.62–3.12)	0.43

Notes: The influence of covariates on the occurrence of extraglandular manifestation was evaluated with a Cox model. The proportional hazard assumption was checked with 2 different methods: graphically by plotting the log(minuslog) curves and by studying the interaction with time. CIE: counterimmunoelectrophoresis. pSS: primary Sjögren’s syndrome. ^a^ Age as continuous variable.

## Data Availability

The data presented in this study are available on request from the corresponding author.

## Supplementary Materials

The following are available online at https://www.mdpi.com/article/10.3390/jcm11010242/s1, Figure S1: Extraglandular manifestations occurring after diagnosis according to the presence or absence of significant sialadenitis in the SSA+CIE− group.
