# Supplementary material for: Discordant Predictions of Extraglandular Involvement in Primary Sjögren’s Syndrome According to the Anti-SSA/Ro60 Antibodies Detection Assay in a Cohort Study
Source: J Clin Med. 2022 Jan 4;11(1):242. doi: 10.3390/jcm11010242 (PMC8745780; doi:10.3390/jcm11010242)
Supplement: Supplementary file 1 [file jcm-11-00242-s001.zip › jcm-1507191-SI.pdf]

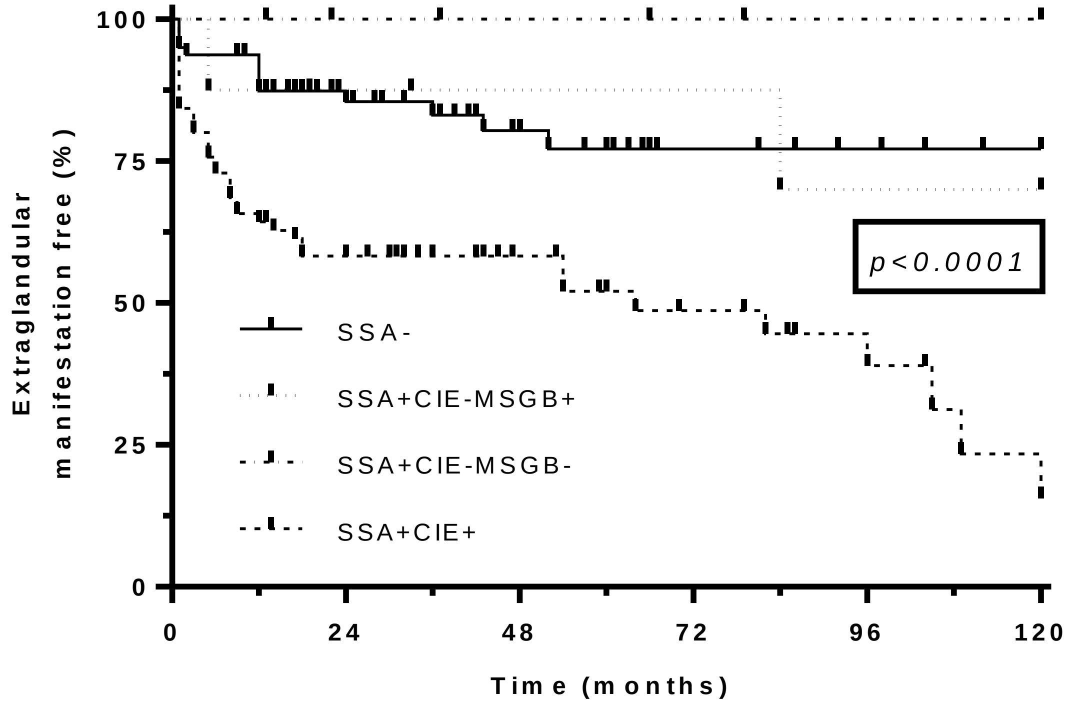

Figure S1. Extraglandular manifestations occurring after diagnosis according to the presence or absence of significant sialadenitis in the SSA+CIE- group. *Notes:* the SSA- group referred to the patients with no anti-SSA antibodies. The SSA+CIE-MGSB+ referred to patients with anti-SSA antibodies in any techniques except for counterimmunoelectrophoresis and with a focus score of ≥ 1 on the MSGB. The SSA+CIE-MGSB- referred to the patients with anti-SSA antibodies in any techniques except for counterimmunoelectrophoresis and with a focus score of < 1 on the MSGB. The SSA+CIE+ referred to the patients with anti-SSA antibodies in counterimmunoelectrophoresis. CIE: counterimmunoelectrophoresis. MGSB: minor salivary gland biopsy. The p-value on the graph represents the results of the comparison between the four curves using the log-rank test.
